# Identifying and modeling the impact of neonicotinoid exposure on honey bee colony profit

**DOI:** 10.1093/jee/toae227

**Published:** 2024-10-22

**Authors:** Miriam Bixby, Sarah K French, Sydney B Wizenberg, Aidan Jamieson, Mateus Pepinelli, Morgan M Cunningham, Ida M Conflitti, Leonard J Foster, Amro Zayed, Maria Marta Guarna

**Affiliations:** Department of Biochemistry and Molecular Biology, University of British Columbia, Vancouver, BC, Canada; Department of Biology, York University, Toronto, ON, Canada; Department of Biology, York University, Toronto, ON, Canada; Department of Biology, York University, Toronto, ON, Canada; Department of Biology, York University, Toronto, ON, Canada; Department of Biochemistry and Molecular Biology, University of British Columbia, Vancouver, BC, Canada; Beaverlodge Research Farm, Agriculture and Agri-Food Canada, Beaverlodge, AB, Canada; Department of Biology, York University, Toronto, ON, Canada; Department of Biochemistry and Molecular Biology, University of British Columbia, Vancouver, BC, Canada; Department of Biology, York University, Toronto, ON, Canada; Department of Biochemistry and Molecular Biology, University of British Columbia, Vancouver, BC, Canada; Beaverlodge Research Farm, Agriculture and Agri-Food Canada, Beaverlodge, AB, Canada

**Keywords:** beekeeping profitability, blueberry pollination, neonicotinoids, risk quotients

## Abstract

Pollination by the European honey bee, *Apis mellifera*, is essential for the production of many crops, including highbush blueberries (*Vaccinum corymbosum*). To understand the impact of agrochemicals (specifically, neonicotinoids, a class of synthetic, neurotoxic insecticides) on these pollinators, we conducted a field study during the blueberry blooms of 2020 and 2021 in British Columbia (B.C.). Forty experimental honey bee colonies were placed in the Fraser Valley: half of the colonies were located within 1.5 km of highbush blueberry fields (“near” colonies) and half were located more than 1.5 km away (“far” colonies). We calculated risk quotients for these compounds using their chronic lethal dietary dose (LDD_50_) and median lethal concentration (LC_50_). Pesticide risk was similar between colonies located near and far from blueberry forage, suggesting that toxicity risks are regionally ubiquitous. Two systemic neonicotinoid insecticides, clothianidin and thiamethoxam, were found at quantities that exceeded chronic international levels of concern. We developed a profit model for a pollinating beekeeper in B.C. that was parameterized by: detected pesticide levels; lethal and sublethal bee health; and economic data. For colonies exposed to neonicotinoid pesticides in and out of the blueberry forage radii, there were economic consequences from colony mortality and sublethal effects such as a loss of honey production and compromised colony health. Further, replacing dead colonies with local bees was more profitable than replacing them with imported packages, illustrating that beekeeping management selection of local options can have a positive effect on overall profit.

## Introduction

Managed honey bee (*Apis mellifera*) colonies are an integral component of agricultural ecosystems worldwide. They provide essential pollination services for several lucrative crops including blueberries (Isaacs and Kirk 2010). Although honey bees are not able to buzz pollinate and efficiently release pollen from the bell-shaped blueberry flower, they are able to effectively transfer pollen from flower to flower via different mechanisms (Hoffman et al. 2018). In addition, the large numbers of bees per colony, over 40,000, that are moved to the desired field make honey bees the most commonly managed pollinator of blueberry crops. While pollinating blueberries, honey bees also collect pollen from a variety of other sources (McAfee et al. 2024). The pollen collected is combined with nectar in the colony for fermentation and storage in the form of bee bread. Although bee bread is a source of nutrients for the colony, it is also a source of environmental contaminants including plant viruses and pesticides (Cunningham et al. 2022, Lee et al. 2023). Canada is the second largest producer of blueberries in the world (Agriculture and Agri-Food Canada 2021). In 2021, Canadian honey bees contributed $280 million worth of production value (90% of the total) to both lowbush blueberries (*Vaccinium augustifolium*) and highbush blueberries (*Vaccinum corymbosum*) (Agriculture and Agri-Food Canada 2022). Highbush blueberries are one of Canada’s most economically important fruit crops, grown on over 12,080 hectares in 2022 (Agriculture and Agri-Food Canada 2023). British Columbia (B.C.) accounts for 95% of the country’s highbush blueberry production, which was 66,472 metric tons in 2022, valued at over $180 million (Agriculture and Agri-Food Canada 2023). Although we do not fully understand which stressors affect blueberry pollinating colonies (McAfee 2024), there are many stressors that honey bees are generally exposed to during commercial pollination including: increasing monocultural landscapes (Potts et al. 2010, Dufour et al. 2020); lengthy transportation of colonies (Pettis et al. 2016, McAfee et al. 2020); aggressive pathogens and pests (Guzman-Novoa et al. 2010, Le Conte et al. 2010); and pesticide use (Mullin et al. 2010, Goulson et al. 2015, Graham et al. 2022, European Food Safety Authority 2023). These stressors interact in complex ways that are only recently beginning to be investigated (French et al. 2024). Pesticide application is an agricultural practice that reduces crop losses but can also present a health risk to pollinating insects (Desneux et al. 2007, Sanchez-Bayo and Goka 2014, Graham et al. 2021). A commonly used class of pesticides known as neonicotinoids, or neonics, disrupt the neuromuscular system of insects (Sandrock et al. 2014, Goulson et al., 2015), effectively controlling unwanted crop pests and resulting in improved crop outcomes (Alsafran et al., 2022). Neonics are systemic insecticides that accumulate in plant pollen and nectar, increasing toxicity risks for pollinating insects, including honey bees (Sandrock et al. 2014, Tosi et al. 2017, Tsvetkov et al. 2017, Tsvetkov and Zayed 2021, Graham et al. 2022). The health impact of these pesticides on honey bees is often studied through the lens of lethality. A common metric of acute toxicity is the median lethal dose (LD_50_), which is the dose of a compound that causes death in 50% of test subjects (Organization for Economic Co-operation and Development 1998). The impact of chronic toxicity is more nuanced, with chronic health impacts including lethal and sublethal effects from chemicals manifesting in the test population after a lengthier pesticide exposure (Tosi and Nieh 2017). The overall impact of the test chemical is identified by comparing the health outcomes of the test chemical-treated group to those of the control group (Organisation for Economic Co-operation and Development 2017). Sublethal impacts are physiological or behavioral effects on a population that survives acute or chronic pesticide exposure (Desneux et al. 2007). These sublethal effects can manifest in a small subset of the honey bees within a colony that may or may not affect outcomes at the colony level or there can be sublethal effects on a significant proportion of the colony, resulting in important changes in colony functionality. Sublethal effects can include changes to honey bee learning and memory (Decourtye et al. 2003); impairments in foraging, hygienic, reproductive, and social behaviors (Morfin et al. 2019, Grout et al. 2020, Tison et al. 2020, Tsvetkov and Zayed 2021); changes to flight orientation, navigation, distance, and velocity (Fischer et al. 2014, Tosi et al. 2017); reduced colony immunity, growth, performance, and productivity (Rondeau et al. 2014, Wood et al. 2018, Chambers et al. 2019); higher queen supersedure rates (Sandrock et al. 2014, Tsvetkov et al. 2017); and gut microbiome dysbiosis (Cunningham et al. 2023).

To develop effective policy and regulation that safeguards pollinator health from both lethal and sublethal pesticide health impacts, the risk from short- or long-term exposure to a particular compound must be quantified. One method is to assign an acute risk quotient (RQ) to specific compounds that relate the concentration of an active ingredient to its LD_50_ (EPA-PMRA-CALDPR 2014). RQs can be compared against national and international pesticide risk thresholds (e.g., levels of concern or trigger values), as outlined by organizations including Health Canada’s Pest Management Regulatory Agency (PMRA), the United States Environmental Protection Agency (US EPA), or the European Food Safety Authority (EFSA). The PMRA, the EPA, and the California Department of Pesticide Regulation (CALDPR) produce joint publications on pesticide risk guidance and thus the EPA thresholds referenced here are joint PMRA-EPA-CALDPR values (EPA-PMRA-CALDPR 2014). The risk thresholds for acute oral exposure for adult honey bees are RQ = 0.4, derived from laboratory studies using test cages (EPA-PMRA-CALDPR 2014), and RQ = 0.2, derived from studies using field colonies (European Food Safety Authority 2013a). Currently, neither the PMRA nor the EPA has an explicit threshold for chronic oral exposure to pesticides for pollinators. However, the risk threshold for chronic oral exposure has been calculated for use by the EFSA where RQ = 0.03, again, derived from studies using field colonies (European Food Safety Authority 2013a). These RQ values may reflect a lower risk tolerance threshold in Europe compared to North America, although both values for acute oral exposure are about tenfold higher than the EFSA chronic oral exposure thresholds. Chronic exposure risks can be quantified as sublethal effects, in relation to a “no observed effects dose,” at and below which no measurable pesticide effects can be detected (United States Environmental Protection Agency 2015, Thompson 2021). Chronic risks can also be quantified as lethal effects, through the chronic lethal dietary dose (LDD_50_) or median lethal concentration (LC_50_), which result in 50% mortality after 10 days of exposure (Organisation for Economic Co-operation and Development 2017). By comparing the acute and chronic RQs for a specific compound to a risk threshold, compounds of concern are identified, and regional environmental management interventions can be effectively implemented to minimize and mitigate pesticide risk to pollinators.

The Fraser Valley, an agriculturally rich region of southwestern British Columbia, produces nearly all of Canada’s highbush blueberries (BC Agriculture in the Classroom Foundation 2024). Honey bee colonies in the Fraser Valley are typically placed in highbush blueberry fields in the spring when blueberry plants are at about 5–10% bloom, and colonies are removed at petal drop several weeks later (Morandin and Law 2021). During the pollination period in any intensive agricultural region, there is an acute and chronic toxicity risk to pollinators from both target pesticide application (applied to protect blueberry crops) and nontarget pesticide application (applied to adjacent nonblueberry crops, see Table 1 for details on B.C. crops and pesticides; McArt et al. 2017, 2017, Graham et al. 2021, 2022, Bishop et al. 2022). It is important to note that pesticide residues on vegetation, soil, and water can remain in ground and surface water as well as after the spray period, or time of seeding with pesticide-coated seeds, and as a result pose a threat to pollinators beyond the target crop bloom. In 2020 and 2021, we measured and identified pesticide compounds and levels in honey bee foraged pollen and nectar from colonies placed near (< 1.5 km away) and far (>1.5 km away) from blueberry fields in this region of B.C. We calculated the acute RQs for each pesticide found in our experimental colonies and compared the RQs to EFSA and EPA risk thresholds to gauge lethal and/or sublethal impacts. We also calculated and compared the chronic RQs to the EFSA threshold for the 2 neonicotinoids in our study that were found at concentrations that posed the highest acute risk to honey bees. Informed by the RQs and risk thresholds, we developed and parameterized a profit model to estimate the economic effect of both lethal and sublethal toxicity from exposure to neonicotinoids on honey bee colony productivity and profit. Estimating beekeeper per colony profit using empirical data provides the beekeeping industry with evidence-based economic values that can support decision-making, and our modeling builds on previous beekeeping profit model estimation (Bixby et al. 2017, 2020, 2021, 2023b).

**Table 1.** The common and genus names of crops grown in British Columbia, and a list of the 20 pesticides detected during our study at T2 and/or T3

Genus	Vaccinium	Fragaria	Rubus	Ribes	Allium		Brassica	Lathyrus	Zea	Solanum	Phaseolus	Vitis
Common	Blueberry (also cranberry)	Strawberry	Raspberry (also blackberry)	Currants/gooseberries	Garlic (also leeks)	Green bunching (also dry bulb) onions	Cole crops (also turnips)	Peas	Corn	Potatoes	Snap beans	Grapes
**Pesticide (*n* instances)**												
Boscalid (33)	X	X	X (X)	X	X	X (X)	X			X	X	X
Chlorantraniliprole (1)	X (X)		X (X)	X		X	X	X	X	X		X
**Clothianidin** (6)									**X**	**X**		**X**
Coumaphos (1)												
Difenoconazole (3)	X	X		X	X (X)	X (X)	X (X)			X		X
Dimethoate (4)	X	X					X	X		X	X	
Fenhexamid (1)	X	X	X (X)	X								X
Flonicamid (4)		X					X (X)	X		X	X	
Fluopyram (47)	X	X	X (X)							Y		X
Flupyradifurone (50)	X	X	X (X)	X			X	X	X	X	X	X
Imidacloprid (30)	X	X	X (X)				X (X)	X		X	X	X
Linuron (21)									X	X		
Mandipropamid (1)					X	X (X)	X			X		
Metconazole (22)	X									X		
Napropamide (1)	X (X)	X	X (X)		X		X (X)					X
Novaluron (2)	X (X)			X			X		X	X		
Omethoate (1)												
Pyraclostrobin (33)	X	X	X (X)	X	X	X (X)	X (X)			X		X
Pyrimethanil (37)	X	X	X (X)		X	X (X)				X		X
**Thiamethoxam (15)**	**X (X)**	**X**	**X**	**X**			**X**		**X**	**X**		

An “X” indicates that a pesticide is generally applied to a given crop, and “(X)” indicates the pesticide is also applied to the congeneric in brackets. Pesticide application data are derived from British Columbia’s guides for crop production and for grapes (Government of British Columbia 2023). *Raphanus* (radish) and *Rheum* (rhubarb) pollen were excluded as they are not treated with these pesticides, and *Humulus* (hops) was excluded due to a lack of data on pesticide applications. See Table S1 for vegetables that were undifferentiated in AAFC maps.

Blueberry growers’ priority is to produce healthy crops of blueberries which requires optimal bee pollination and the effective management of unwanted pests and diseases. The effect of toxicity exposure on bees from pesticides reduces both the efficacy of bee pollination and the overall health of honey bee colonies, jeopardizing the beekeeping industry and limiting the availability of healthy pollinators. Blueberry grower management decisions are inextricably linked with honey bee colony health and beekeeper profit. This research was motivated by a need to identify synergies that exist between these 2 industries and the opportunity to support both industries simultaneously. This is the first colony-level profitability analysis of the effects of pesticides on honey bees. By determining the pesticide exposure risk for honey bees and the resulting economic impact for the industry in this region, we can provide beekeepers and policy-makers with empirical data to optimize bee health and beekeeping profits and to support a vibrant pollination-dependent blueberry sector.

## Methods

### Study Design, Field Exposure, and Sampling

**Fig. 1.**
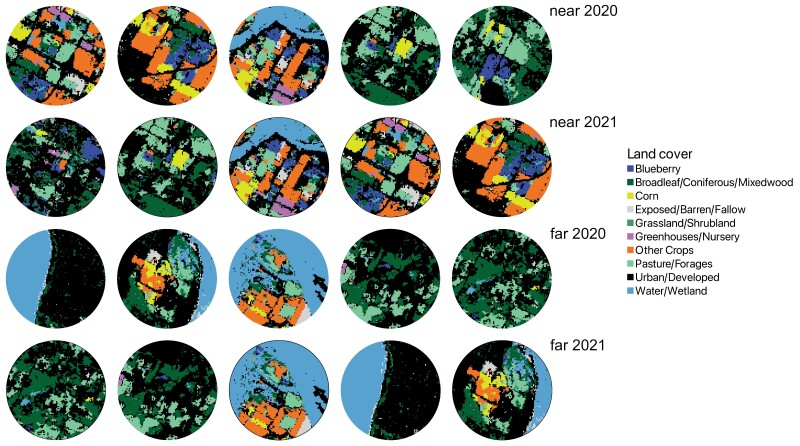
Land cover within 1.5 km of 20 sites in the Fraser Valley of British Columbia, indicating potential sources of crop-related honey bee (*Apis mellifera*) colony exposure to agrochemicals in sites within 1.5 km of highbush blueberry (*Vaccinum corymbosum*) (“near”) and sites greater than 1.5 km away from highbush blueberry (“far”). Sites were sampled in 2020 or 2021. The Other Crops category includes barley, orchards, other berry/crops/vegetables, peas, potatoes, sod, and vineyards.

Study colonies were located in the Fraser Valley of B.C. during the pollination periods for highbush blueberry (as described in French et al. 2024, McAfee et al., 2024). Briefly, during the beekeeping seasons of 2020 and 2021, 4 honey bee colonies (i.e., 1 apiary) were placed at each of 20 sites; the replication of sites allowed us to generalize patterns across different landscapes. The colonies were sourced from local beekeepers and no record was kept as to whether the 2020 colonies were used again in 2021. The use of 4 colonies allowed us to have at least 3 colonies in the event of colony loss during the experiment. This study was part of a larger study across Canada where the design included that colonies were located in 5 sites near and 5 sites far from crops, in 2 subsequent years. Thus, each year, colonies were placed at 10 sites in or adjacent to highbush blueberry fields (generally ≈0 km away but always less than 1.5 km away; hereafter referred to as “near” sites), and at 10 sites that were at least 1.5 km away from highbush blueberry fields (hereafter referred to as “far” sites, Fig. 1). Sites were located at least 3 km apart, such that foraging resources were assumed to be distinct (Richardson et al. 2023) as honey bees are expected to forage near their colony (Balfour and Ratnieks 2017). At the beginning of the experiment, colonies contained single brood chambers, a 1-year-old queen, and were clear of noticeable signs of disease or other health issues. The land cover surrounding each site, within a 0.5, 1.5, and 2.5 km radius, was identified using the 2020 and 2021 Annual Crop Inventory from Agriculture and Agri-Food Canada (AAFC, Agriculture and Agri-Food Canada 2021; see Fig. 1 for land cover at 1.5 km). We used these spatial data to identify land cover types possibly related to the detection of certain agrochemicals in our samples and to relate to pollen genera that were detected in our samples. Our near sites were selected using fields with prominent highbush blueberry cover, in cooperation with landowners. We selected far sites based on the lack of noticeable blueberry cover, based on visual observations and landowner knowledge of the surrounding land cover. However, based on the land cover data from AAFC that was released after our field seasons had concluded, and based on bee bread analysis, the far sites also had some potential blueberry cover (see McAfee et al. 2024). Although we cannot determine the accuracy of the AAFC data at this small scale, the proportion of land covered by blueberry at far sites was substantially smaller than that at near sites.

Colonies were sampled twice for pesticides during the pollination season: after colonies were placed at experimental sites when >50% of highbush blueberry plants were in bloom in the region (Time Point 2, T2); and at the end of the blooming period (Time Point 3, T3). Bloom was assessed by the crop farmers at each site, who communicated this information to us and the beekeepers. Bee bread/pollen and nectar were collected at each of the 2-time points. Freshly deposited pollen (i.e., lightly packed and dry) was collected from each colony using a fresh disposable stir stick and transferred to a falcon tube. Nectar (uncapped honey) was collected with a new 1-cc syringe per colony (with no needle) and transferred to a centrifuge tube that was immediately placed in the dark. All samples were stored on dry ice in the field and then moved to an ultralow −80°C freezer. Before analysis, samples from the 4 colonies of an apiary were pooled; 8 g of pollen and 12 ml of nectar were analyzed at the Agriculture and Food Laboratory (University of Guelph, Guelph, Ontario; ISO/IEC 17025 accredited). A multiresidue pesticide analysis was performed to quantify 239 agrochemicals using standard methods (Payá et al. 2007, Canadian Food Inspection Agency 2008, French et al. 2024, see Supplementary Dataset for Limit of Detection and Limit of Quantification values). The pesticides detected in nectar and pollen within the honey bee colonies possibly reflected those applied to both the target crop that the colonies are rented to pollinate, and to nontarget crops in the surrounding fields. Pollen subsamples were analyzed in-house using multilocus metabarcoding to identify pollen grains to the genus level (Wizenberg et al. 2023). We compared the genus names of the pollen to their [potential] crop cover equivalents in the landscape, to determine which floral resources were available to the colonies. In order to generalize patterns within the pollination period for highbush blueberry, which in our case spanned approximately 1 month, as well as between years, we pooled the data from T2 and T3, and 2020 and 2021, keeping the individual data points raw and unchanged.

### RQ and Thresholds

To understand the empirical short-term risk that pesticides posed to honey bees, we calculated an acute RQ (RQ_acute_) for each pesticide based on the concentration of the compound detected in pollen and nectar, the estimated consumption of pollen and nectar by bees, and the median lethal dose (LD_50_) for acute oral exposure to bees (EPA-PMRA-CALDPR 2014, United States Environmental Protection Agency 2015, Thompson 2021, Rondeau and Raine 2022, Odemer et al. 2023, French et al. 2024). The LD_50_ of each pesticide was determined from the literature (see French et al., 2024). The dietary RQ_acute_ of the combination of pollen and nectar was calculated for each pesticide compound detected at a site, for each time point separately and assuming bees consume 140 mg of nectar and 9.6 mg of pollen per day (EPA-PMRA-CALDPR 2014, United States Environmental Protection Agency 2015, Thompson 2021, Rondeau and Raine 2022, Odemer et al. 2023, French et al. 2024):


$$\begin{array}{l} RQ_{acute}=\frac{\begin{array}{c} \left(residue\;in\;nectar\left(\mu \;g\;kg^{-1}\right)\times 140\times 10^{-6}\;kg\;bee^{-1}\right)\;+\left(residue\;in\;pollen\left(\mu \;g\;kg^{-1}\right)\times \;9.6\times 10^{-6}\;kg\;bee^{-1}\right) \end{array}}{acute\;oral\;LD_{50}\left(\mu \;g\;bee^{-1}\right)} \end{array}$$

These acute RQs represent the relative lethal toxicity of pesticide residues to honey bees. RQ = 1 indicates that half of a honey bee population exposed to this compound will not survive. We assumed that the death of half of the bees in a test population would be directly equivalent to the mortality of half the bee population in colonies (European Food Safety Authority 2023), resulting in a decrease in colony strength. We compared RQs between sites near (<1.5 km away) and far (>1.5 km away) from highbush blueberry for each pesticide. We used a generalized linear model in R (R Core Team 2022) with a Gamma error structure, where RQs plus a small constant were log-transformed. Posthoc comparisons were made using *emmeans* (version 1.8.7; Lenth 2023), using Bonferroni’s adjustment. We also calculated the sum of RQ_acute_ for all pesticides present at each site and time point, which gave us a total RQ_acute_ where effects were considered additive (see Traynor et al. 2016, Graham et al. 2022, French et al. 2024), allowing us to compare pesticide risk between near and far sites. This comparison was done using a generalized linear mixed-effects model (*lme4* package, version 1.1-34; Bates et al. 2015) with a Gamma error structure, where RQs were log-transformed RQs, and site was included as a random effect. In visualizations of our RQs, we included the acute risk threshold for field colonies (i.e., RQ = 0.2; European Food Safety Authority 2013a) to gauge the likelihood of lethal (and sublethal) effects in a comparable field setting to those measured by the EFSA.

Given that the duration of active blueberry pollination in the Fraser Valley is several weeks and bee bread is consumed over time, bees can be chronically exposed to contaminants in bee bread. Thus, we also quantified the long-term (chronic) risk of exposure to key pesticides during the pollination period. Specifically, we examined 2 neonicotinoids, clothianidin and thiamethoxam, that are commonly used in agricultural settings such as the Fraser Valley (Reeves 2022, Government of Canada 2024). Clothianidin is not currently registered for use on blueberries in the Fraser Valley while Thiamethoxam is registered for use on highbush blueberries; however, there may be an impact from off-label uses as well. We calculated RQ_chronic_ for these 2 compounds, using literature LC_50_ and LDD_50_ values where bees are exposed to a pesticide for 10 days (European Food Safety Authority 2013b, 2016). The dietary RQ_chronic_ was then calculated as:


$$\begin{array}{l} RQ_{chronic}=\frac{\begin{array}{c} \left(residue\;in\;nectar\left(\;\mu \;g\;kg^{-1}\right)\times 140\times 10^{-6}\;kg\;bee^{-1}\right)\;+\left(residue\;in\;pollen\left(\;\mu \;g\;kg^{-1}\right)\times 9.6\times 10^{-6}\;kg\;bee^{-1}\right) \end{array}}{chronic\;10\;day\;oral\;LC_{50}\;or\;LDD_{50}\left(\mu \;g\;bee^{-1}\;day^{-1}\right)} \end{array}$$

In visualizations of our RQ_chronic_ values, we included the chronic risk threshold (i.e., RQ = 0.03; European Food Safety Authority 2013a) to gauge the likelihood of lethal (and sublethal) effects from exposure to 2 neonicotinoids in a comparable field setting to those measured by the EFSA.

### Profit Model

We developed a profit model for a beekeeper who rents colonies for commercial blueberry pollination in British Columbia’s Fraser Valley. Following previous profit modeling (Bixby et al. 2017, 2020, 2021, 2023b) and recent survey data (Bixby et al. 2023a), we know that for a subset of surveyed beekeepers, 70% of beekeeping revenue in B.C. accrues from honey and pollination sources (with honey accounting for 60% and pollination 10%). Bee production is also a revenue source for beekeepers; however, in a recent study less than 1% of surveyed Canadian beekeepers engaged in bee sales as a revenue source, and as a result, we have focused our profit function on honey and pollination revenues, like other profit models (Bixby et al. 2017, 2023b). In this model, we represent a beekeeper who produces honey and rents colonies for commercial pollination, much like many beekeepers in British Columbia. This model allows us to estimate profit using regionally relevant pollination and honey data and to explore the impact of pesticide toxicity at the colony level. Beekeeper profit was total revenue, which includes honey and pollination revenue, less total cost, which includes the cost to maintain a commercially pollinating colony, and any replacement costs in case of colony mortality. The profit equation (through honey revenue) explicitly considered any direct changes in honey production because of pesticide toxicity effects and the equation also included a health variable that accounted for any indirect effects from pesticide toxicity. The health variable was parameterized using our RQs calculated for neonicotinoids sampled from colonies in the Fraser Valley test sites and fulfills a similar function to other honey bee health impact variables in profit modeling literature such as deteriorating health or overwintering losses due to parasite infestations (Bixby et al. 2017, 2021). The health variable in this model represented colony changes such as behavioral and other physiological impairments (*h*_*it*_ = 0 for no other pesticide impact and *h*_*it*_ = 1 for lethal impact) (Tosi et al. 2017, Morfin et al. 2019) and captured both lethal and sublethal effects that were not directly related to honey production, for example reproduction, brood development and health (Morfin et al. 2019), as well as foraging and flight (Fischer et al. 2014, Tosi et al. 2017). Both sublethal and lethal effects can be direct (honey production alone) or indirect (behavioral/physiological). The RQ_chronic_ values were compared to a chronic threshold of RQ = 0.03 (European Food Safety Authority 2013a). If the RQ for a sample of honey bee pollen/nectar that was exposed to a pesticide in our study was greater than 0.03, the EFSA chronic threshold above which there are negative health effects (European Food Safety Authority 2013b, 2016), we assumed that there would likely be some colony mortality as well as sublethal effects on the surviving colonies in that apiary. As the first study to model profit impacts from pesticide exposure on highbush blueberries at the colony level, we chose to simplify the nuanced and complex effects of pesticide exposure within a honey bee colony. To this end, we rely on the simplifying assumption that the lethal and sublethal effects of exposure to a toxic compound can impact the profit function in 3 ways: (i) a direct reduction in the colony’s honey output and resulting revenue; (ii) changes in colony behavior and physiology represented by the health variable and resulting in indirect effects that further decreased productivity (revenue); and (iii) an additional cost of colony replacement in the case that the colony did not survive. We will, henceforth, refer to the behavioral and physiological health changes as indirect effects, contrasting the direct effect of changes in honey production. Studies point to honey production decreasing by between 7% and 30% after several weeks of pesticide exposure (Wood et al. 2018, Chambers et al. 2019). Also, there is evidence that chronic pesticide impacts and synergistic effects can be delayed in manifesting and affect bee and colony performance months after the exposure (Rondeau et al. 2014, Straub et al. 2019). When the colony presented with sublethal effects in early summer, we assumed that honey production would decrease by the upper limit of 30%, as the colony experienced the effects of pesticide poisoning for the entire season. Whereas, if sublethal effects manifested in the fall, we assumed honey production would be impacted by the lower limit of 7% to account for a shorter-term impact.

Even if there is no tangible manifestation of symptoms visible to the beekeeper after pesticide exposure in the short term, a colony that showed lethal or sublethal effects in the fall was unlikely to be at optimal health throughout the season (Wu et al. 2011, Sanchez-Bayo and Goka 2014). To account for the deterioration of a colony’s honey productivity leading up to colony failure (death) from pesticide exposure that manifested in the fall, we assumed honey production decreased that season by 18.5% (the mid-way point between sublethal impacts in the summer and the fall). This is a simplifying assumption in our modeling that allows us to make calculations in spite of the uncertainty involved in the time-lag effect of pesticide exposure (Rondeau et al. 2014). Since a colony that demonstrated exposure effects later in the fall was likely to be at suboptimal health throughout the season due to pesticide toxicity, the indirect effects were captured by the health variable that was parameterized as greater than zero in the fall, implying a nonzero pesticide health impact. Whether the colony died in the summer or the fall, the beekeeper will pay a similar colony replacement cost as the beekeeper will either make a split (same labor cost regardless of time of year) or wait to purchase a package in the spring at the market price. Our colony-level model was run through various scenarios representing different lethal and sublethal outcomes for the honey bee colony following pesticide exposure. Depending on the time of year when pesticide toxicity impacts manifested, there was greater or lesser impact on a beekeeping operation’s revenue as varying amounts of honey production and indirect toxicity effects impacted profit. The model’s key assumptions are shown in Table 2. The revenue and cost data used in the model were collected from BC beekeepers in a recent survey (Bixby et al. 2023b).

**Table 2.** Profit model assumptions about lethal and sublethal pesticide effects on honey bees (*Apis mellifera*) and colony replacement

	Time of year (*t*) beekeeper first identifies symptoms					
	Early summer			Fall		
Pesticide effects	None	Sublethal	Lethal	None	Sublethal	Lethal
Colony replacement	No	No	Yes	No	No	Yes
Health *h*_*t*_(0, 1)	*h* = 0	0 ≤ *h* ≤ 1	*h* = 1	*h* = 0	0 ≤ *h* ≤ 1	0 ≤ *h* ≤ 1

Profit model assumptions about the effects of highly toxic pesticide exposure for a colony through the health variable *h*_*t*_(0, 1) and whether colony replacement is necessary given the timing of sublethal or lethal colony symptom manifestations.

#### Profit Model Scenarios

Colony profit with *no* indirect pesticide impacts on the colony (*h*_*it*_ = 0):

$\pi_{i}=[\left(\left(1-h_{it}\right)\;\left(P_{ih}*\;Q_{ih}\right)\right)+\left(RFbl_{i}\right)-Cop_{i}]$ *where h*_*it*_ = 0 *so profit simplifies to*


$$\begin{array}{l} \pi_{i}=[\left(\left(P_{ih}*\;Q_{ih}\right)+\left(RFbl_{i}\right)\right)-Cop_{i}] \end{array}$$

1. Pesticide exposure manifests in indirect colony impacts in **early summer**.
  a. Sublethal effects (0 < *h*_*it*_ < 1)

$$\begin{array}{l} \pi_{i}=[\left(\left(1-h_{it}\right)\;\left(P_{i}*\;Q_{i}\right)\right)+\left(RFbl_{i}\right)-\left(Cop_{i}\right)] \end{array}$$

b. Lethal effects (*h*_*it*_ = 1)

$$\begin{array}{l} \pi_{i}=[\left(\left(1-h_{it}\right)\;\left(P_{i}*\;Q_{i}\right)\right)+\left(RFbl_{i}\right)-\left(Cop_{i}+C_{rep}\right)] \end{array}$$

where h_it_ = 1 so profit simplifies to

$$\begin{array}{l} \pi_{i}=[\left(RFbl_{i}\right)-\left(Cop_{i}+C_{rep}\right)] \end{array}$$

1. Pesticide exposure manifests in indirect colony impacts in the **fall**.
  a. Sublethal effects (0 < *h*_*it*_* < 1)*

$$\begin{array}{l} \pi_{i}=[\left(\left(1-h_{it}\right)\;\left(P_{i}*\;Q_{i}\right)\right)+\left(RFbl_{i}\right)-\left(Cop_{i}\right)] \end{array}$$

b. Lethal effects (0 < *h*_*it*_* < 1)*

$$\begin{array}{l} \pi_{i}=[\left(\left(1-h_{it}\right)\;\left(P_{i}*\;Q_{i}\right)\right)+\left(RFbl_{i}\right)-\left(Cop_{i}+C_{rep}\right)] \end{array}$$

where π_*i*_ was the yearly profit for colony *i*, *h*_*it*_(0, 1) was the health variable for colony *i* that indicates the degree of other indirect (behavioral/physiological) colony health impacts that affected colony performance from pesticide exposure. *P*_*i*_ was the price per unit kg of honey received by the beekeeper, *Q*_*i*_ was the quantity of honey in kg from colony *i* sold by the beekeeper, and *RFbl*_*i*_ was the rental fee paid to the beekeeper for blueberry pollination services from colony *i*. A colony with sublethal pesticide impact may have exhibited a myriad of symptoms such as less honey production (Wood et al. 2018, Chambers et al. 2019), which will be reflected in *Q*_*i*_, while other behavioral and physiological changes such as reproductive, brood and flight changes would be captured as indirect effects in the health parameter, *h*_*it*_(0, 1). *Cop*_*i*_ was the total operating cost the beekeeper paid to manage colony *i* throughout the season and *Crep* was the cost paid by the beekeeper to replace the dead colony in case of a lethal pesticide impact. Beekeepers are typically equipped with comprehensive best practice management information when dealing with diseases and parasites such as *Nosema* spp. and *Varroa destructor*. However, guidance on pesticide exposure is primarily focused on prevention (Morandin and Law 2021), resulting in few postexposure treatment options. Best management practices postexposure are often limited to minimizing any ongoing exposure and waiting to see if the remaining bees recover and survive (University of Georgia 2023). As a result, in our model, there were no additional specific pesticide treatment costs.

Table 3 lists the initial parameter values used in the profit model. Price per kilogram of honey was $18.56 ($8.42/lb) and per colony honey production was 27 kg (59 lbs), the average price and quantity for a group of recently surveyed Canadian beekeepers who rented their colonies for commercial pollination in B.C. (Bixby et al. 2023b). Honey production for colonies exhibiting sublethal effects was 19 kg (41 lbs) when pesticide effects manifested in the summer and 25 kg (55 lbs) when pesticide effects manifested in the fall, 30% and 7% respectively less than the full honey crop (Wood et al. 2018, Chambers et al. 2019). The fee for a pollinating honey bee colony to pollinate blueberries was $124/colony, which was the average rental fee paid to a sample of Canadian beekeepers renting bees for blueberry pollination in B.C. in 2022/2023 (BC Sector Data 2023 data available upon request). The cost for a beekeeper to maintain and support a honey bee colony that gets rented out for blueberry pollination was $400, the average cost paid in B.C. by a group of beekeepers who rented their colonies for commercial pollination in 2021/2022 (Bixby et al. 2023b). Beekeepers choose to replace lost colonies with imported packages or by making splits within their operation and adding a queen. The colony replacement cost with a package was $240 and the cost of making a split (labor cost $10) and buying a queen ($45) was $55 (Bixby et al. 2023b).

**Table 3.** Initial profit model assumptions for honey bee colonies (*Apis mellifera*)

Pesticide impact	*P*_*i*_ ($/kg)	*Q*_*i*_ (kg)		*RFbl*_*i*_ ($/col)	*Cop*_*i*_ ($)	*Crep*_*i*_ ($)	
						Split	Pckg.
		Summer	Fall				
None	$18.56	27	27	$124	$400	n/a	n/a
Sublethal	$18.56	19	25	$124	$400	n/a	n/a
Lethal	$18.56	0	22	$124	$400	$55	$240

Profit model assumptions given: no pesticide effects; sublethal effects; or lethal effects for the initial parameterizations of honey price (*P*_*i*_), honey production (*Q*_*i*_), highbush blueberry (*Vaccinum corymbosum*), pollination rental fee (*RFbl*_*i*_), colony operating cost (*Cop*_*i*_), and colony replacement cost (*Crep*_*i*_). (*P*_*i*_ is the honey price in $/kg produced by a colony *i* in a season, *Q*_*i*_ is the honey produced by colony *i* in a season (kg/colony), *RFbl*_*i*_ is the pollination rental fee in $ accruing to colony *i* for a pollination contract, *Cop*_*i*_ is the colony operating cost in $ to manage colony *i* in a season and *Crep*_*i*_ is the colony replacement cost to replace colony *i*.)

### Sensitivity Analysis

The effects of pesticide exposure and the resulting economic impacts on a honey bee colony is a complex process with many variables including colony resilience, market pricing, and beekeeper management. As a result, in our sensitivity analysis, we investigated a range of honey production impacts (Sagili and Burgett 2011, Wood et al. 2018, Chambers et al. 2019) where honey output was parameterized at 10% or 3 kg (6 lbs), 50% or 14 kg (30 lbs), and 90% or 24 kg (53 lbs) of a full honey crop or 27 kg (59 lbs) for a pollinating colony depending on the season. The analysis also explored the effect of a reduced blueberry pollination rental fee to 50% ($62) and 75% ($93) of the full rental fee ($124) because of pesticide exposure and resulting suboptimal colony size and strength overtime, impacting a colony’s pollination rental fee potential (Wood et al. 2018, Chambers et al. 2019, Leska et al. 2021). Profit was also calculated for a beekeeper who produced an in-house queen to lower colony replacement costs such that the cost for a beekeeper using a split and an in-house queen to replace a lost colony was $28.75, where $10 was for labor and $18.75 was for queen production (Table 4; Bixby et al. 2020). Profit in the sensitivity analysis was calculated for each new parameter value described above, ceteris paribus (with all other variables remaining in their initial parameterizations as seen in Table 3).

**Table 4.** Honey bee (*Apis mellifera*) colony profit model assumptions for the sensitivity analysis

Pesticide impact	*P*_*i*_ ($/kg)	*Q*_*i*_ (kg)		*RFbl*_*i*_ ($/col)	*Cop*_*i*_ ($)	*Crep*_*i*_ ($)	
		Summer	Fall			Split	Pckg.
None	$18.56	27	27	$124	$400	n/a	n/a
Sublethal	$18.56	3,14	24	$62, $93	$400	n/a	n/a
Lethal	$18.56	0	14	$62, $93	$400	$28.75	$240

Profit model assumptions given no pesticide effects, sublethal effects, or lethal effects for the sensitivity analysis parameterizations of honey price (*P*_*i*_), honey production (*Q*_*i*_), highbush blueberry (*Vaccinum corymbosum*), pollination rental fee (*RFbl*_*i*_), colony operating cost (*Cop*_*i*_), and colony replacement cost (*Crep*_*i*_). (*P*_*i*_ is the honey price in $/kg produced by a colony *i* in a season, *Q*_*i*_ is the honey produced by colony *i* in a season (kg/colony), *RFbl*_*i*_ is the pollination rental fee in $ accruing to colony *i* for a pollination contract, *Cop*_*i*_ is the colony operating cost in $ to manage colony *i* in a season and *Crep*_*i*_ is the colony replacement cost to replace colony *i*.)

## Results

### Pesticide Exposure

**Fig. 2.**
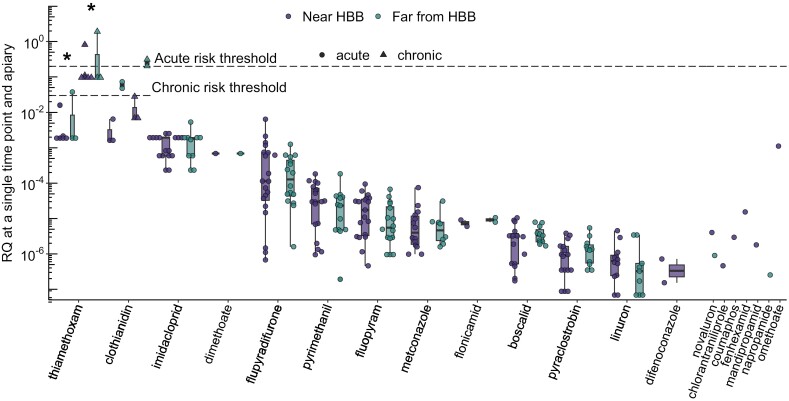
Risk quotients (RQs) for 20 pesticide compounds found in the study colonies. Honey bee (*Apis mellifera*) colonies were located either in highbush blueberries (*Vaccinum corymbosum*) (near HBB) or more than 1.5 km away (far from HBB). Data points represent RQs from Time Points 2 and 3, and the study years 2020 and 2021, and are replicated for thiamethoxam and clothianidin to consider both acute and chronic RQs. The box plots show the median, the 25th and 75th quantiles, and nonoutlier minima/maxima as whiskers; asterisks indicate significant differences between near and far RQ_acute_ values for a pesticide. The dashed lines represent risk thresholds for honey bees’ acute (RQ = 0.2) and chronic (RQ = 0.03) oral exposure to pesticides.

**Fig. 3.**
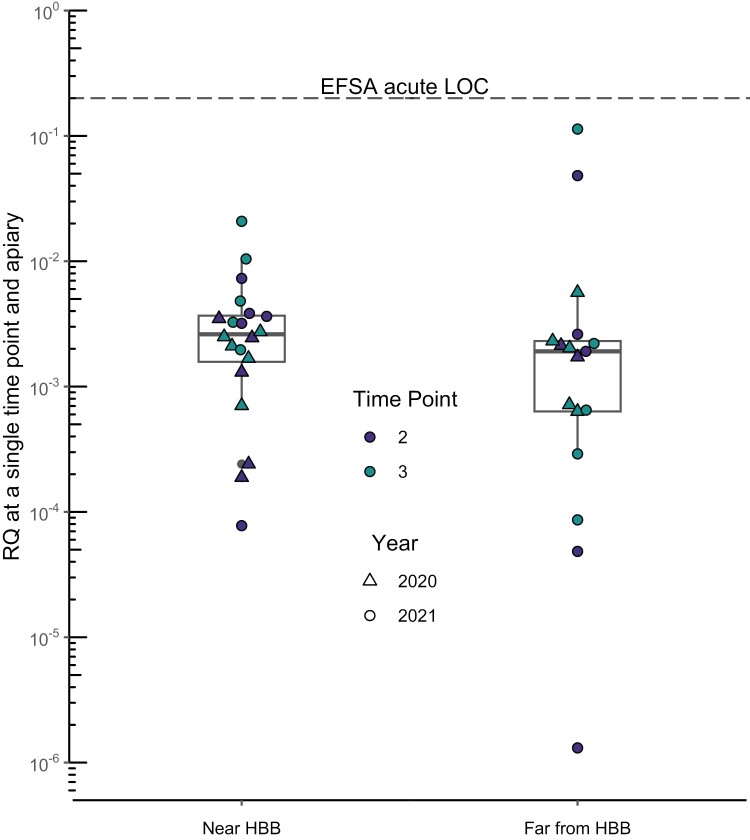
Total risk quotients (RQs) representing all pesticide compounds detected in the 20 honey bee (*Apis mellifera*) apiaries. Each apiary was located at one of 20 different sites, 10 of which were located within 1.5 km of highbush blueberries (*Vaccinum corymbosum*) (near HBB), and 10 of which were located more than 1.5 km away (far from HBB). Data points represent RQs from Time Points 2 and 3, and the study years 2020 and 2021. The box plots show the median, the 25th and 75th quantiles, and nonoutlier minima/maxima as whiskers. The dashed line represents the risk threshold for honey bees’ acute (RQ = 0.2) oral exposure to pesticides.

The colonies in our study were exposed to a total of 21 pesticide compounds after being placed either near (<1.5 km away) or far (>1.5 km away) from highbush blueberry (see list of pesticides in Table 1), with 14 being detected at more than 1 site and time point (Fig. 2). RQs are listed for each compound in Supplementary Dataset (SD). No RQ_acute_ exceeded the acute threshold of RQ = 0.2 (European Food Safety Authority 2013a). Two neonicotinoids, clothianidin and thiamethoxam, generally had higher RQs in sites located far from highbush blueberry than those located in or near highbush blueberry (*t* = 24 and *t* = 5.4, for clothianidin and thiamethoxam, respectively, and df = 223 and *P* < 0.001 in both cases). All other pesticides had similar RQs between near and far sites (*t* = −0.54–0.30, df = 223, *P* = 1 in all cases; the compound mefenacet was excluded as relevant LD_50_s could not be determined). Clothianidin and thiamethoxam also had the highest RQs overall and were detected: in pollen sampled in 2020 from colonies near to highbush blueberry; in both nectar and pollen sampled in 2021 from colonies both near and far from highbush blueberry; during and at the end of the pollination period (T2 and T3). The RQ_chronic_ for thiamethoxam exceeded the chronic threshold of RQ = 0.03 (European Food Safety Authority 2013a) at 8 separate sites, with 3 of these sites also having clothianidin RQ_chronic_ that exceeded this threshold (Fig. 2). There were no significant differences in total dietary RQ_acute_ between sites near and far from highbush blueberry (*t* = −0.028, df = 33, *P* = 0.98; Fig. 3), a result consistent with other research on pesticide exposure and pollination (Pettis et al. 2013, Graham et al. 2022).

### Profit Modeling

**Fig. 4.**
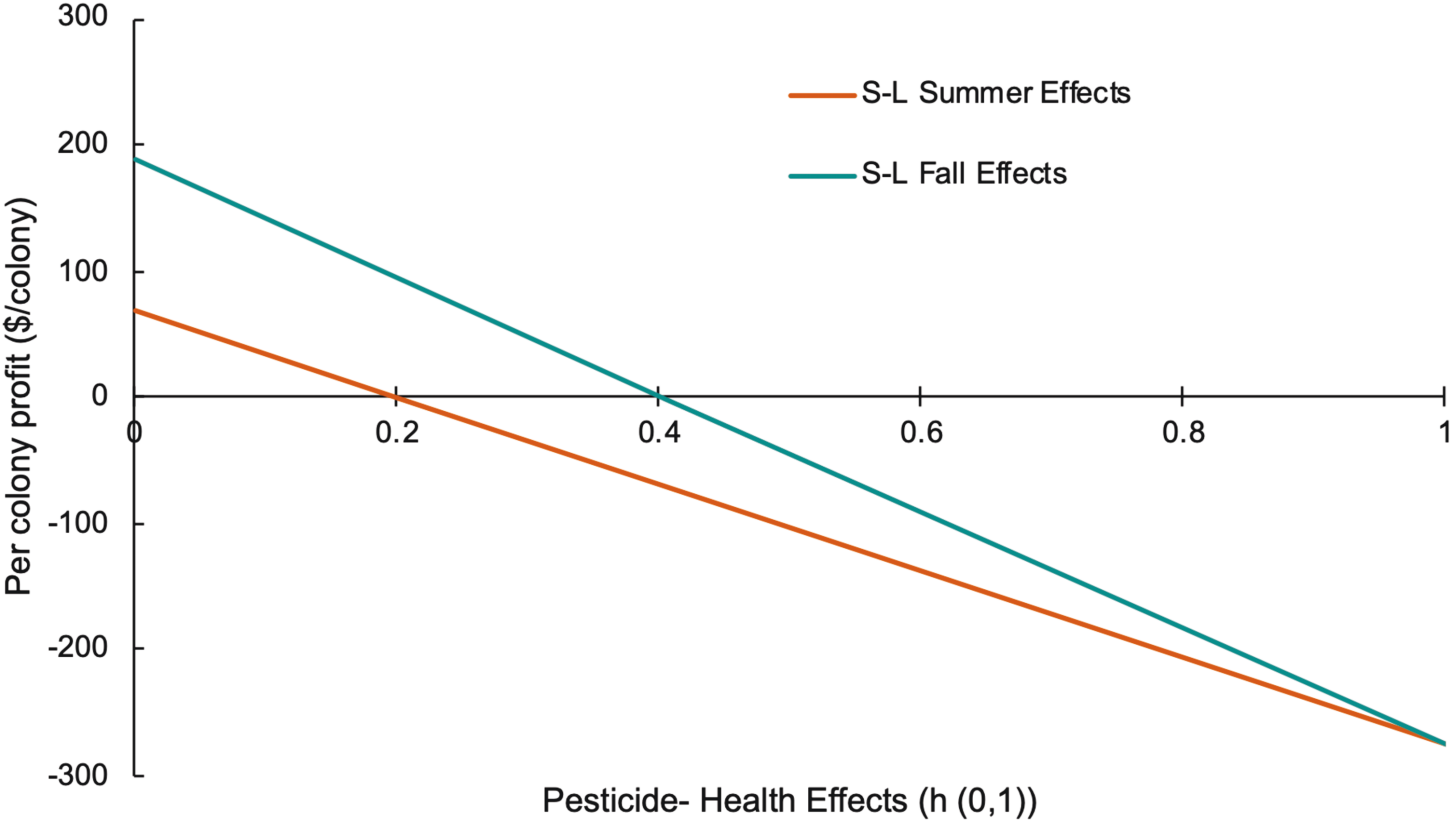
Per colony profit as a function of the colony health variable that captures the sublethal (S-L) impacts from pesticide exposure to honey bee (*Apis mellifera*) colonies, given direct honey production impacts.

When there is no pesticide exposure effect on a colony, per colony profit was $220.78 (Table 5, Fig. 4). For a colony with reduced honey production from systemic insecticide exposure in the early summer after the blueberry pollination period, given the initial parameterizations with between a 7% and 30% decrease in honey production from pesticide toxicity, profits fell to between $69.22 and −$276.00, depending on the severity of the other indirect sublethal health impacts as represented by the health variable (*h*_*it*_; Fig. 4). When a colony died (*h*_*it*_ = 1) in the early summer, following blueberry pollination, profits fell to between −$331.00 and −$516.00 when the lost colony was replaced by a split or a package, respectively. When the beekeeper observed sublethal toxicity effects in the colony in the fall, per colony profit ranged from $187.00 to −$276.00 depending on the severity of the other indirect health impacts (Fig. 4). If the colony died in the fall, profits ranged from −$111.84 to −$516.00 when packages were used for replacement and between $73.16 to −$331.00 when splits were used.

**Table 5.** Honey bee (*Apis mellifera*) colony profit model results for the initial parameterizations given the indirect health effects, direct honey production effects replacement strategy and timing of colony symptom manifestation

Indirect effects (health variable *h*_*it*_)	Direct effects (honey production) and replacement strategy						
		Early summer			Fall		
	None	Sublethal	Lethal		Sublethal	Lethal	
	n/a	n/a	Package	Split	n/a	Package	Split
*h*_*it*_ (zero*π*)^a^	n/a	0.2005	n/a	n/a	0.4040	<0	0.1810
Per colony profit range (as a function of *h*_*it*_)							
*π* (*h*_*it*_ = 0)	$220.78	$69.22	n/a	n/a	$187.10	−$111.84	$73.16
*π* (*h*_*it*_ = 1)	n/a	−$276.00	−$516.00	−$331.00	−$276.00	−$516.00	−$331.00

Net colony loss is shown in grey.

^a^These results are the values for the health variable when per colony profit is equal to zero under the different pesticide effects and colony replacement scenarios.

### Sensitivity Analysis

When greater variability in honey production was factored into the model, the results predictably showed higher profits for more honey production and lower profits for less honey production, regardless of the time of year (Table S2). When the colony was still able to produce 90% of the full honey crop with no other sublethal indirect health effects identified in the early summer, per colony profit was $170.26; however, profits fell to a low of −$331.00 with lethal impacts in the fall and split bee replacement. Also, when the honey bee colony was too small or weak to accrue an average pollination rental fee due to longer-term chronic pesticide effects, profits fell as rental income fell resulting in a range of profits falling from $220.78 with no pesticide effects to −$578.00 with lethal effects and package replacement (Table S3). When a beekeeper replaced a colony loss with a split and used a queen from within their operation, the cost of replacement fell, and profits rose from a low of −$331.00 with lethal effects and split replacement with a purchased queen to a maximum of $99.41 with an in-house queen (Table S4).

## Discussion

Colonies in the Fraser Valley of British Columbia are exposed to systemic insecticides during blueberry bloom, whether they are commercially pollinating highbush blueberries or are outside the typical honey bee foraging radius from highbush blueberries, as has also been observed in recent pollination-pesticide studies (McArt et al. 2017, Graham et al. 2022). In our study, honey bee colonies were exposed to similar levels of total acute dietary risk (an additive measure reflecting the risk of all pesticides) whether they were placed near to (<1.5 km) or far from (>1.5 km) prominent highbush blueberry fields. Specifically, 13 pesticide compounds were detected in both near and far colonies. However, when the risk of individual pesticide compounds at these different locations was assessed, 2 neonicotinoids—clothianidin and thiamethoxam—had greater levels of overall risk in far colonies. While these 2 compounds did not exceed the acute threshold for dietary risk, they did in some cases exceed the risk threshold for chronic exposure (European Food Safety Authority 2013a). Both compounds have been shown to impact colonies through a multitude of lethal and sublethal mechanisms (e.g., Decourtye et al. 2003, Fischer et al. 2014, Sandrock et al. 2014, Tosi et al. 2017, Wood et al. 2018, Cunningham et al. 2023, Liu et al. 2023) and are generally found in agricultural settings such as the Fraser Valley. Thiamethoxam is registered for use on highbush blueberries in Canada (Morandin and Law 2021), as well as several other crops (Table 1, Table S1). Clothianidin, however, is not registered for use on highbush blueberries in Canada but is used primarily to control insects in vegetables such as corn and potatoes (Table 1, Table S1). Both corn and potatoes were in proximity to near (<1.5 km away from highbush blueberry) and far (>1.5 km away from highbush blueberry) study colonies (Fig. 1) but are not typically reliant on honey bee pollination. However, since both neonicotinoids are highly water soluble, clothianidin that is applied to corn and/or potatoes could enter the surrounding water systems and get subsequently taken up by blueberry plants, nearby wildflowers, or weeds where honey bees may then be exposed (Tsvetkov et al. 2017). The combination of data yielded from pollen analysis, the identification of surrounding crop cover, and knowledge of common pesticide applications (Tables 1, 6, and S1), suggested a likely source of exposure to thiamethoxam via applications to highbush blueberry, at least for the near colonies. However, the source of clothianidin exposure was less clear as this is not a compound used to treat Fraser Valley’s highbush blueberry fields (Tables 1, 6, and S1). Corn (*Zea*), potato (*Solanum*), and/or vineyard (*Vitis*) cover were present in the landscape surrounding the sites with clothianidin present (Table 6). However, these genera were not detected in pollen samples, likely because corn is wind-pollinated, grapes are self- and wind-pollinated, and honey bees do not generally pollinate potatoes (Wheelock et al. 2016, Buchanan et al. 2017, Kratschmer et al. 2019).

**Table 6.** Sites near (<1.5 km) or far (>1.5 km) from highbush blueberry with detections (X) of thiamethoxam or clothianidin in the pollen/nectar of honey bee (*Apis mellifera*) colonies

Site	Thiamethoxam	Clothianidin	Pollen genera detected		0.5 km radius	1.5 km radius	2.5 km radius
Near	X RQ > 0.03		◦ Brassica ◦ Malus ◦ Prunus	◦ Raphanus ◦ Rubus ◦ Vaccinium	• Blueberry • Corn • Vegetables* • Potatoes • Barley		+ Cranberry
	X RQ > 0.03		◦ Brassica ◦ Malus ◦ Prunus	◦ Raphanus ◦ Rubus (not T2) ◦ Vaccinium	• Blueberry • Corn • Vegetables*	+ Berry* + Potatoes	+ Crops* + Fruits*
	X RQ > 0.03	X RQ > 0.03	◦ Brassica ◦ Malus ◦ Prunus	◦ Raphanus ◦ Rubus ◦ Vaccinium	• Blueberry • Vegetables* • Potatoes • Vineyards	+ Corn + Berry* + Barley	+ Peas
	X RQ > 0.03	X RQ < 0.03	◦ Brassica ◦ Malus ◦ Prunus	◦ Raphanus ◦ Rubus ◦ Vaccinium	• Blueberry • Corn • Berry*	+ Vegetables* + Potatoes + Crops*	
	X RQ > 0.03	X RQ < 0.03	◦ Brassica ◦ Malus ◦ Prunus	◦ Raphanus (not T3) ◦ Rubus ◦ Vaccinium	• Blueberry • Corn • Berry* • Potatoes	+ Vegetables*	+ Hops + Crops*
Far	X RQ > 0.03	X RQ > 0.03	◦ Brassica ◦ Malus (T2) ◦ Prunus	◦ Rheum (T3) ◦ Rubus ◦ Vaccinium	• Blueberry	+ Vineyards + Orchards	+ Corn + Cranberry + Berry*
	X RQ > 0.03		◦ Brassica ◦ Malus ◦ Prunus	◦ Raphanus ◦ Rubus ◦ Vaccinium	• Blueberry • Corn • Potatoes	+ Berry* + Vegetables*	
	X RQ > 0.03		◦ Brassica ◦ Malus ◦ Prunus	◦ Raphanus ◦ Rubus ◦ Vaccinium	• Blueberry • Corn • Vegetables* • Potatoes	+ Berry*	

The chronic RQs are listed as greater or less than the chronic threshold of RQ = 0.03. Pollen genera possibly related to crop cover include *Brassica* (cruciferous vegetables), *Malus* (apple orchards), *Prunus* (prune orchards), *Raphanus* (radish), *Rheum* (rhubarb), *Rubus* (raspberries, blackberries), and *Vaccinium* (blueberries, cranberries). Crop cover was identified within a 0.5, 1.5, and 2.5 km radius from colonies: • crops are found across scales but listed once; + crops are not found at lesser scales; * undifferentiated by AAFC.

When a honey bee colony is exposed to one or both neonicotinoids clothianidin and thiamethoxam, whose RQ values exceed the EFSA chronic level of concern, there are direct and indirect effects that could be both sublethal and lethal and result in decreased colony profit. In cases of compound stressor interactions, effects could be multiplied (Johnson et al. 2013). Our profit modeling shows that lethal and sublethal effects of pesticide exposure in honey bee colonies manifest as lost productivity through direct and indirect pathways and ultimately result in decreased profit. The earlier a colony presents with pesticide-induced health effects, the greater the impact on profit as production is impacted throughout the entire season. In the event of colony mortality, the method of colony replacement is also an important indicator of profitability. When a beekeeper can replace lost colonies with less expensive splits, as opposed to packages, and use an in-house queen instead of a purchased queen, profits are higher. Exposure to stressors that impact pollinators can have an important economic effect on individual beekeeping operations and on the industry. It is important to be transparent about the use of survey data and the accompanying simplifying assumptions that are used to support the parameterization of our economic modeling. Survey data is critical to understanding the beekeeping industry as it is one of the only sources of apicultural data available in Canada; however, survey data relies on nonvalidated beekeeper responses. The sensitivity analysis mitigates some of the risks of using this data and making these assumptions, but there should always be follow-up studies to support this modeling.

Pesticide toxicity, in addition to other stressors, has led to beekeeper hesitancy to send bees into blueberry pollination resulting in pollination limitations for some Canadian crops. When a crop is pollination-limited, there is a supply shortage of pollinating animals like honey bees such that additional pollination would result in increased crop yield. For highbush blueberry crops, this limitation resulted in a pollinator deficiency in 94% of sampled areas in one study (Reilly et al. 2020). The scarcity of adequate pollinators for blueberry crops in British Columbia is the result of direct health impacts from agricultural practices and other stressors on pollinating managed and wild bees (Mullin et al. 2010, Potts et al. 2010, Dufour et al. 2020, Graham et al. 2022, European Food Safety Authority 2023). Blueberry production is a critical agricultural industry in Canada and requires a strong, healthy supply of honey bee colonies (Agriculture and Agri-Food Canada 2021, 2022, 2023). To support this industry as well as Canadian beekeeping, the economic implications of pesticide toxicity in highbush blueberry pollination must be validated by additional studies and translated into policy and regulation that protects and supports beekeepers and their bees. By creating a sustainable beekeeping industry, blueberry grower demand for thriving, pollinating honey bee colonies will be met, and these same policies and regulations will ultimately also protect blueberry growers by ensuring adequate pollination and consumers by optimizing fruit quality and quantity.

## Supplementary data

Supplementary data are available at *Journal of Economic Entomology* online.

toae227_suppl_Supplementary_Material

toae227_suppl_Supplementary_Tables_S2-S4

toae227_suppl_Supplementary_Tables_S1
