# Supplementary material for: Identifying and modeling the impact of neonicotinoid exposure on honey bee colony profit
Source: J Econ Entomol. 2024 Oct 22;117(6):2228–41. doi: 10.1093/jee/toae227 (PMC11682944; doi:10.1093/jee/toae227)
Supplement: toae227_suppl_Supplementary_Tables_S2-S4 [file toae227_suppl_supplementary_tables_s2-s4.docx]

**Supplementary Table S2.** Sensitivity analysis results: honey production.^[[1]](#footnote-1)^ The range of colony profit as a function of variable honey output.

|  | | | | ***Per colony profit range: π f(h_it_)*** | |
| --- | --- | --- | --- | --- | --- |
| **Honey (lbs)** | **Time** | **Initial Effects** | ***h_it_ (*Zero-*π)*** | ***π (h_it_=0)*** | ***π (h_it_=1)*** |
| 59lbs (initial) | n/a | None | n/a | $220.78 | n/a |
| 6lbs | Early Summer | Sub-lethal | <0 | -$225.48 | -$276.00 |
| 30lbs | Early Summer | Sub-lethal | <0 | -$23.40 | -$276.00 |
| 53lbs | Fall | Sub-lethal | 0.3815 | $170.26 | -$276.00 |
| 30lbs | Fall | Lethal | <0 (package) | -$263.40 | -$516.00 |
| 30lbs | Fall | Lethal | <0 (split) | -$78.40 | -$331.00 |

**Supplementary Table S3.** Sensitivity analysis results: pollination rental fee. The range of colony profit as function of variable pollination rental fees.

|  | | | | ***Per colony profit range: π f(h_it_)*** | |
| --- | --- | --- | --- | --- | --- |
| **Fee** | **Time** | **Initial Effects** | ***h_it_ (*Zero-*π)*** | ***π (h_it_=0)*** | ***π (h_it_=1)*** |
| $124 (initial) | n/a | None | n/a | $220.78 | n/a |
| $62 | Early Summer | Sub-lethal | 0.0209 | $7.22 | -$338.00 |
| $62 | Early Summer | Lethal (*h_it_=1)* | n/a | n/a | -$578.00 (pckg) |
| $62 | Early Summer | Lethal (*h_it_=1)* | n/a | n/a | -$393.00 (split) |
| $62 | Fall | Sub-lethal | 0.2701 | $125.10 | -$338.00 |
| $62 | Fall | Lethal | <0 | -$173.84 | -$578.00 (pckg) |
| $62 | Fall | Lethal | 0.0276 | $11.16 | -$393 (split) |
| $93 | Early Summer | Sub-lethal | 0.1107 | $38.22 | -$307.00 |
| $93 | Early Summer | Lethal (*h_it_=1)* | n/a | n/a | -$547.00 (pckg) |
| $93 | Early Summer | Lethal (*h_it_=1)* | n/a | n/a | -$362 (split) |
| $93 | Fall | Sub-lethal | 0.3371 | $156.10 | -$307.00 |
| $93 | Fall | Lethal | <0 | -$142.84 | -$547.00 (pckg) |
| $93 | Fall | Lethal | 0.1043 | $42.16 | -$362.00 (split) |

**Supplementary Table S4.** Sensitivity analysis results: in-house queen for replacement split.^[[2]](#footnote-2)^ The range of colony profit as a function of variable colony replacement costs.

|  | | |  | ***Per colony profit range*** | |
| --- | --- | --- | --- | --- | --- |
| **Cost split** | **Time** | **Initial Effects** | ***h_it_ (*Zero-*π)*** | ***π (h_it_=0)*** | ***π (h_it_=1)*** |
| $55 (initial) | Early Summer | Lethal | n/a | n/a | -$331.00 |
| $28.75 | Early Summer | Lethal | n/a | n/a | -$304.75 |
| $55 (initial) | Fall | Lethal | 0.1810 | $73.16 | -$331.00 |
| $28.75 | Fall | Lethal | 0.2460 | $99.41 | -$304.75 |

.

1. *We only consider scenarios in which honey is produced (there is no honey produced when a colony has experienced lethal pesticide effects in early summer).*  [↑](#footnote-ref-1)
2. *We only consider lethal effects as this is a scenario that addresses colony replacement following mortality.* [↑](#footnote-ref-2)
