# Supplementary material for: Identifying and modeling the impact of neonicotinoid exposure on honey bee colony profit
Source: J Econ Entomol. 2024 Oct 22;117(6):2228–41. doi: 10.1093/jee/toae227 (PMC11682944; doi:10.1093/jee/toae227)
Supplement: toae227_suppl_Supplementary_Tables_S1 [file toae227_suppl_supplementary_tables_s1.docx]

| **Genus** | **Asparagus** | **Daucus** | **Apium** | **Cucurbita** | **Anethum** | **Solanum** | **Lactuca** | **Pastinaca** | **Capsicum** | **Spinacia** | **Assorted**  **specialty vegetables** | **Corylus** | **Assorted tree fruit orchards** |
| --- | --- | --- | --- | --- | --- | --- | --- | --- | --- | --- | --- | --- | --- |
| **Common** | **Asparagus** | **Carrots** | **Celery** | **Cucurbits** | **Dill** | **Eggplant** | **Lettuce** | **Parsnips** | **Peppers** | **Spinach** |  | **Hazelnut orchards** |  |
| **Pesticide** |  |  |  |  |  |  |  |  |  |  |  |  |  |
| boscalid |  | X |  | X |  |  | X |  | X | X | X |  | X |
| chlorantraniliprole |  |  | X | X |  | X | X |  | X | X | X | X | X |
| **clothianidin** |  | **X** |  | **X** |  | **X** |  | **X** | **X** |  |  |  |  |
| coumaphos |  |  |  |  |  |  |  |  |  |  |  |  |  |
| difenoconazole |  | X |  | X |  |  |  |  | X |  | X |  | X |
| dimethoate | X |  | X |  |  |  | X |  | X |  | X | X | X |
| fenhexamid |  |  |  |  |  |  |  |  |  |  |  |  | X |
| flonicamid |  |  | X | X |  | X | X |  | X | X | X |  | X |
| fluopyram |  |  |  |  |  |  |  |  |  |  |  |  | X |
| flupyradifurone |  |  | X | X |  | X | X |  |  | X |  |  | X |
| imidacloprid |  | X | X | X |  | X | X | X | X | X | X | X |  |
| linuron |  | X | X |  | X |  |  | X |  |  |  |  |  |
| mandipropamid |  |  |  | X |  |  | X |  |  | X | X |  |  |
| metconazole |  |  |  |  |  |  |  |  |  |  |  | X | X |
| napropamide |  |  |  | X |  |  |  |  | X |  | X |  |  |
| novaluron |  |  | X |  |  |  |  |  | X |  | X |  | X |
| omethoate |  |  |  |  |  |  |  |  |  |  |  |  |  |
| pyraclostrobin |  | X | X | X |  |  |  |  |  | X | X |  | X |
| pyrimethanil |  |  |  |  |  |  |  |  |  |  |  |  | X |
| **thiamethoxam** |  |  |  |  |  | **X** |  |  | **X** | **X** | **X** |  |  |

**Supplementary Table S1.** The common and genus names of crops grown in British Columbia, and a list of the 20 pesticides detected during our study at T2 and/or T3. An ‘X’ mark indicates that a pesticide is generally applied to a given crop. Pesticide application data are derived from British Columbia’s Production Guides for crops (Gov. B.C. 2023), as well as the BC Tree Fruit Production Guide (BCFGA 2023). No pollen from these genera was detected in samples, and AAFC land cover maps did not differentiate among these crops.
